# Dynamic URP: Revisiting Urethral Retro-Resistance Pressure for Contemporary Sphincter-Targeted Therapy

**DOI:** 10.3390/diagnostics15151855

**Published:** 2025-07-23

**Authors:** Nicole Fleischmann

**Affiliations:** White Plains Hospital Center, New York, NY 10601, USA; nfleischmann@wphospital.org

**Keywords:** external urinary sphincter, stress urinary incontinence, urethral retro-resistance pressure, pelvic floor dysfunction, guarding reflex, functional urology, neuromuscular control, urethral function testing

## Abstract

This paper introduces a new conceptual framework for interpreting urethral retro-resistance pressure (URP) as a dynamic, intra-procedural tool—ΔURP—for evaluating external urethral sphincter (EUS) engagement during injection therapy. With renewed interest in therapies that directly target the EUS, there is a critical need for real-time functional feedback at the site of action. This conceptual review re-examines URP in the context of emerging EUS-targeted treatments—such as bulking agents, regenerative injections, and neuromodulatory interventions—and proposes a dynamic model (ΔURP) to measure changes in sphincteric resistance as a functional biomarker during intervention. We review the anatomical, neurophysiological, and histological features of the EUS complex; trace the clinical rise and decline of URP; and compare its utility to conventional diagnostic tools. ΔURP, defined as the change in URP from baseline, is explored as an objective measure of EUS function. We outline its potential applications in guiding therapy, evaluating response, and standardizing outcomes across treatments. Conventional urodynamic measures fail to isolate distal sphincter function. In contrast, URP directly challenges the EUS and, when combined with imaging or procedural tools, may provide real-time feedback on sphincter engagement. When reframed as a dynamic, motion-based readout, URP may fill a critical gap in procedural urology—offering a physiologic signal of therapeutic engagement during EUS-targeted interventions. ΔURP has the potential to revive and repurpose a once-abandoned method into a clinically actionable biomarker for next-generation continence care.

## 1. Introduction

Stress urinary incontinence (SUI) affects nearly half of all women during their lifetime and remains one of the most under-treated conditions in women’s health [1,2]. While traditional therapies and diagnostic frameworks have focused on the mid-urethra and bladder neck, emerging treatments target the distal part of the urethral closure mechanism, which has been largely excluded from both therapeutic and evaluative paradigms.

This paper proposes a new conceptual framework: to redefine urethral retro-resistance pressure (URP)—a once abandoned measure—as a dynamic biomarker of sphincter engagement at the level of the external urethral sphincter (EUS). Termed ΔURP, this approach emphasizes functional change rather than static pressure values. While the paper does not present new clinical data, it introduces a model aligned with the physiologic goals of modern EUS targeted interventions.

Historically, continence has been evaluated through tests that emphasize urethral mobility, leak point pressure, and passive resistance—reflecting a focus on structural integrity and pressure transmission. However, these approaches do not account for the persistent symptoms or variable outcomes we see—particularly in women whose anatomy appears “normal” on examination or imaging, or who meet the objective criteria for cure yet continue to report bothersome symptoms. Increasingly, investigators are recognizing that continence may ultimately depend on the neuromuscular closure mechanism—proposing that unless therapies specifically address this most distal element—both in anatomical location and reflexive function—we may be failing to treat a primary location for urine leakage [3,4].

Despite its central role in urinary continence, the EUS remains poorly assessed by current diagnostic tools [5]. Interventions aimed at enhancing EUS function—including stem cell injections, neuromodulatory therapies and regenerative bulking agents—demand real-time physiologic confirmation of EUS engagement [6,7]. Conventional measures like urethral pressure profilometry (UPP) and leak point pressure (LPP) provide indirect assessments and fail to capture the dynamic properties of the EUS.

This review proposes a modern reinterpretation of ΔURP as a dynamic, intra-procedural tool to evaluate functional responsiveness at the site of therapeutic action. The goal is to reposition ΔURP not as a diagnostic test but as a real-time biomarker during targeted therapy. In doing so, we aim to close the gap between anatomical changes and physiologic outcome—offering a model for integrating structural and functional information during contemporary minimally invasive interventions that treat female SUI.

## 2. Functional Anatomy of the External Urethral Sphincter Complex

The female external urethral sphincter (EUS) is a composite structure responsible for urethral closure during bladder filling and physical stress. Although traditionally described as a single striated muscle, the EUS is more accurately understood as a sphincteric complex consisting of both intrinsic and extrinsic components. These elements differ in their anatomical relationships to the urethra, their roles in continence, and their patterns of neural control.

### 2.1. Structural Composition

The EUS comprises three distinct, striated muscle components:

- Sphincter urethrae (rhabdosphincter): A circular muscle embedded within the urethral wall, forming the intrinsic component responsible for tonic closure during bladder filling.
- Compressor urethrae: A strap-like muscle arising bilaterally from the ischiopubic rami and coursing ventrally over the urethra to compress it against the anterior vaginal wall.
- Urethrovaginal sphincter: A sling-like muscle encircling both the urethra and the anterior vaginal wall at the distal urethra, compressing the opening of the vagina and urethra against the pubic bone.

These skeletal muscles, often grouped under the umbrella of “the EUS,” can be anatomically and functionally distinguished. Each plays a specific role in the continence mechanism, and recognizing these distinctions is essential for diagnostic precision and effective targeted therapy.

**Figure 1:**
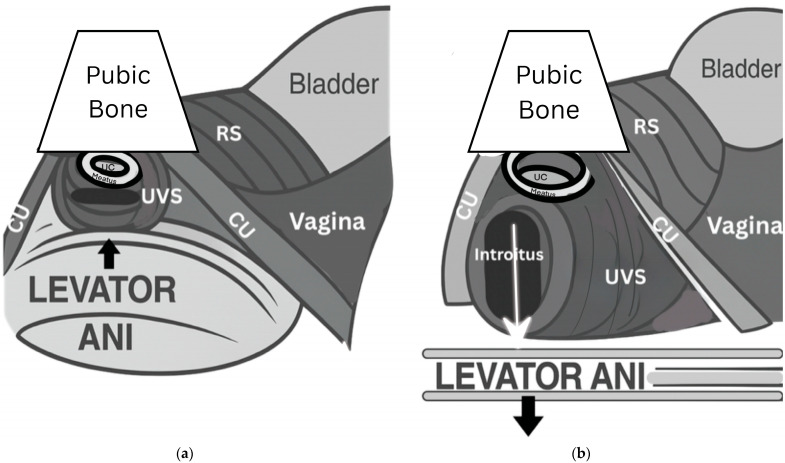
Functional Urethral Anatomy. The distal anatomy changes depending on the functional state (resting/holding and relaxing/voiding). Dynamic closure mechanism of distal urethra. The left image shows that the sphincter complex closes by compression onto the urethral crest and opens (right image to void). CU = compressor urethrae; UC = urethral crest; RS = rhabdosphincter; UVS = urethrovaginal sphincter. (**a**) Holding State: compression of the external sphincter complex during active holding state; note levator musculature in elevated position (**b**) Voiding State: inhibition of all components of EUS complex, including levator musculature for voiding.

The rhabdosphincter is intrinsic to the urethra and provides baseline continence through tonic contraction. In contrast, the compressor urethrae and urethrovaginal sphincter are extrinsic pelvic floor muscles, phasically recruited during acute increases in intra-abdominal pressure (IAP) [8], Rather than behaving as a simple purse-string mechanism, these muscles exert vertical compression onto the urethral crest—a cavernosal-like structure located at the 6 o’clock position of the distal posterior urethra [9]. Continence is supported further by the sealant properties of smooth muscle, vasculature, and mucosa. This distal sphincter complex operates synergistically with the levator ani muscles, particularly the puborectalis muscle, to maintain the high-pressure zone of the vagina [10]. Figure 1 demonstrates the proposed mechanism for pelvic floor coordination with urethral holding and voiding functional states.

### 2.2. Histological and Fiber Type Composition

The external urethral (EUS) complex exhibits a diverse tissue composition that supports its multivariate functional role. The rhabdosphincter is composed predominantly of type I slow-twitch muscle fibers, providing the sustained, low-level contraction required for resting continence. The compressor urethrae and urethrovaginal sphincter contain a higher proportion of type II fast-twitch fibers, facilitating brief, forceful contractions during acute stress events such as coughing or sneezing [5,11].

Unlike in males—where the EUS is more distinctly ring-shaped—the female EUS is organized as a dynamic, layered complex. Striated and smooth muscle layers aggregate in various orientations and degrees of overlap. These fibers do not form a uniform sphincter but instead constitute a sphincteric zone, in which closure is achieved through the coordinated activity of multiple muscular structures [12]. This arrangement allows for precise modulation of urethral resistance in response to both voluntary and reflexive inputs.

### 2.3. Neurologic Control of the EUS Complex

All components of the external urethral sphincter (EUS) complex receive motor input via branches of the pudendal nerve (S2–S4), but emerging evidence suggests important distinctions in their motor pathways and recruitment patterns [13].

The rhabdosphincter is innervated by motor neurons arising from Onuf’s nucleus, a specialized cell group in the ventral horn of the sacral spinal cord. These neurons display a tonic firing pattern and high fatigue resistance, aligning with the rhabdosphincter’s role in maintaining continence during restful bladder filling [14]. The continuous output operates on a default motor program, always active unless inhibited by the descending control centers—the pontine micturition center (PMC) and periaqueductal gray (PAG) [15].

In contrast, the compressor urethrae and urethrovaginal sphincter are located beneath the perineal membrane and are innervated by branches of the deep perineal nerve, a division of the pudendal nerve. These muscles are recruited phasically in response to abrupt rises in IAP (e.g., coughing or sneezing) but are also voluntarily engaged [9]. Their fast-twitch fiber composition and distinct reflex latency profiles suggest separate neuromuscular control from the rhabdosphincter. This divergence mirrors the embryological origins: the rhabdosphincter arises from mesenchyme surrounding the urogenital sinus and integrates directly into the urethral wall. In contrast, the compressor urethrae and urethrovaginal sphincter develop from perineal mesenchyme—similar to other components of the pelvic floor musculature [16].

The understanding that the EUS is a complex with distinct neurophysiologic parts that function as a unit is a critical development in the emergence of an accurate conceptual framework for urethral function [17]. It helps to explain some of the complexities of EUS behavior. For instance, the term “guarding reflex” is often used to describe the quick action of the EUS to contract during provocative movements. However, unlike the strap muscles, there is little evidence that rhabdosphincter participates in a phasic reflex arc during the acute elevation of intra-abdominal pressure (IAP) [12]. 

Although the rhabdosphincter is not phasically recruited, it can develop pathologically heightened tone in the setting of chronic sympathetic activation, stress, or maladaptive motor programming. This is not a guarding reflex in the traditional sense, but an up-regulation of default motor output that can create physical changes in the muscular architecture. This is best exemplified in Fowler’s Syndrome, where women with urinary retention exhibit prolonged motor unit potentials on needle EMG localized to the striated urethral sphincter, despite preserved detrusor function [18]. Women with SUI are shown to have structural changes in the rhabdosphincter as well [19].

## 3. Clinical and Diagnostic Implications

### 3.1. The Role of the External Urethral Sphincter (EUS) in Passive Continence and Voiding

Functional urination remains an area of active investigation, and the external urethral sphincter (EUS) complex has emerged as a critical element in both continence and voiding. While the precise mechanism of voiding initiation has been difficult to capture in human studies, animal models provide insight. The micturition reflex appears to begin with descending bulbospinal inhibition of the sympathetic tone, followed by suppression of tonic output from Onuf’s nucleus via the pontine micturition center (PMC), resulting in reduced urethral resistance [20,21,22,23]. As Watanabe et al. (2014) demonstrated, the urethra actively opens—rather than passively distends—at the onset of micturition through coordinated neuromuscular relaxation [24]. Failure to fully release tension within the distal sphincter, particularly from the compressor muscles, can create a functional bottleneck that impedes flow despite detrusor contraction—resulting in dysfunctional voiding patterns.

Deindl et al. (1998) linked dysfunctional voiding to inappropriate activation of striated pelvic floor muscles during detrusor contraction, underscoring the importance of neuromuscular timing and coordination [25]. Poor relaxation of the extrinsic muscles may directly correlate with rhabdosphincter weakness. Moreover, imaging studies show that continent women demonstrate stronger and more synchronized urethral function than those with SUI [19].

The EUS also plays a central role in maintaining passive continence. In 1980, Rud et al. reported that the striated sphincter accounts for approximately one-third of resting urethral pressure, with the remainder derived from smooth muscle tone and the vascular bed [26]. Later models, including those by DeLancey et al., reinforced the EUS’s contribution to both tonic pressure and reflexive closure [27]. Some investigators, however, have emphasized that maximal urethral pressure occurs at the mid-urethra—where synthetic slings are placed—arguing that the striated sphincter plays a limited role [28].

Radiologic studies counter this assumption. Santiago et al. (2015) [29] found that women with stress urinary incontinence (SUI) exhibited enlarged rhabdomyosphincter dimensions on 3D ultrasound, suggesting altered tone or compensatory overactivity. These findings support the notion that the EUS is dynamically involved in both continence and voiding and may be an under-recognized contributor to dysfunction [29].

### 3.2. The EUS Dual-Component System Model

Viewing the EUS as a dual-component system—an intrinsic tonic rhabdosphincter coupled with extrinsic phasic compressor muscles—offers a more physiologic model of continence. Dysfunction can be attributed to failure of rhabdosphincter inhibition, resulting in urinary retention or dysfunctional voiding, or failure of the phasic system to activate during provocative stress maneuvers, contributing to SUI. Although SUI is often attributed to urethral hypermobility or fascial insufficiency, these structural models fail to distinguish between active and reflexive deficits. As stated by Hokanson et al., “The dynamic function of the urethra is likely mediated by reflexively activated striated muscles, which include the compressor urethrae and the urethrovaginal sphincter. These muscles contract in response to stress events to augment urethral closure pressure” [30].

### 3.3. Quantifying EUS Function

Despite its central role in both continence and coordinated voiding, the EUS has historically been difficult to assess in isolation. Conventional urodynamic tests—such as leak point pressure (LPP), and urethral pressure profiles (UPP)—offer general information about urethral resistance but lack the spatial resolution or temporal specificity to distinguish the dynamic function of the distal sphincter complex.

Electromyography (EMG) is informative for identifying neuropathic patterns such as denervation but shows poor correlation with clinical severity of SUI [18]. In addition, EMG remains of limited utility due the variability of electrode placement and artifactual signals.

Maximum urethral closure pressure (MUCP), a component of UPP, is commonly used to infer outlet strength. By convention, a value over 20 cm H20 implies adequate urethral function. However, MUCP measurements are not sensitive to treatment effects—remaining unchanged after midurethral slings and bulking agents—even when patients report clinical improvement [31]. Weber (2001) concluded that MUCP lacks diagnostic reliability for SUI in women, largely due to the absence of standard protocols and its static nature [32].

Urethral pressure reflectometry (UPR) is a promising technique for capturing real-time striated sphincter behavior. Unlike UPP, which relies on static or pulled-pressure metrics, UPR measures cross-sectional area and urethral pressure during stress maneuvers, allowing for assessment of the reflexive closure response. Saaby (2014) introduced the concept of abdominal-to-urethral pressure impact ratio (APIR) as a functional metric for assessing sphincter response under elevated IAP [33]. This marks a shift from static to dynamic models of urethral performance.

**Figure 2:**
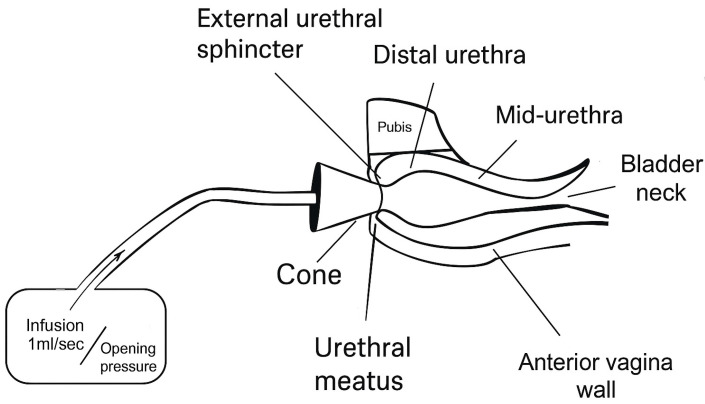
Urethral retro-resistance pressure (URP) was introduced as a noninvasive method to quantify EUS function through continuous retrograde fluid infusion into the urethral meatus using a sealed “plug” or “cone” at the opening of the urethrovaginal sphincter. Unlike catheter-based urodynamic studies, which often introduce artifacts and discomfort, URP allows for measurement of sphincteric opening pressure without disrupting tone.

Urethral retro-resistance pressure (URP), first described by Slack et al. (2004) [34], is a direct method for assessing distal urethral resistance. The technique involves occluding the urethral meatus with a soft cone and infusing saline retrograde at a controlled rate until flow though the closure mechanism is observed. The opening pressure reflects the resistance of the distal EUS complex [34]. Unlike catheter-based methods, URP avoids artificial stimulation of sphincter reflexes that might confound accuracy as well as improve patient comfort [35]. Figure 2 demonstrated how URP measurement is obtained.

Early studies confirmed that URP correlates with other validated measures such as MUCP and LPP. URP is significantly lower in women with SUI [36,37,38,39]. However, URP has been criticized for poor prediction of leakage severity. In addition, Roderick et al. (2009) demonstrated that URP did not change significantly after midurethral sling procedures [40]. Due to poor reproducibility and lack of standardized thresholds, URP was never adopted for diagnostic use.

However, the critiques of URP as a static, one-time measurement may have overlooked its latent value. Reframing URP as a dynamic procedural biomarker—where change from baseline (ΔURP) is used to assess real-time therapeutic engagement—could revive its relevance. When combined with continuous imaging, URP could serve as a feedback mechanism to verify procedural efficacy during bulking, regenerative, or neuromodulatory interventions. When paired with cystoscopic or ultrasound guidance, dynamic URP may provide actionable feedback to confirm mechanical coaptation or neuromuscular activation in situ, closing the gap between procedural intent and physiologic response.

### 3.4. Therapeutic Applications of Dynamic URP

This structural framework has direct implications for both treatment and diagnostics. Point pressure measurements or static assessments may fail to capture the EUS’s dynamic states. Because the EUS is treated in the neutral (not voiding) state, URP is ideal for measuring resting urethral resistance and assessing therapeutic effect.

Even early critics of URP acknowledged that it offered a direct measure of resting resistance. Petros argued that URP could not diagnose mechanical failure at ligamentous or fascial support structures but might represent a useful window into the urethra’s biomechanical behavior—what he called “a limited (but perhaps significant) measure of urethral elasticity” [41]. This view supports the reinterpretation of URP not as a standalone diagnostic but as a procedural feedback tool for assessing sphincter strength during therapy.

**Figure 3:**
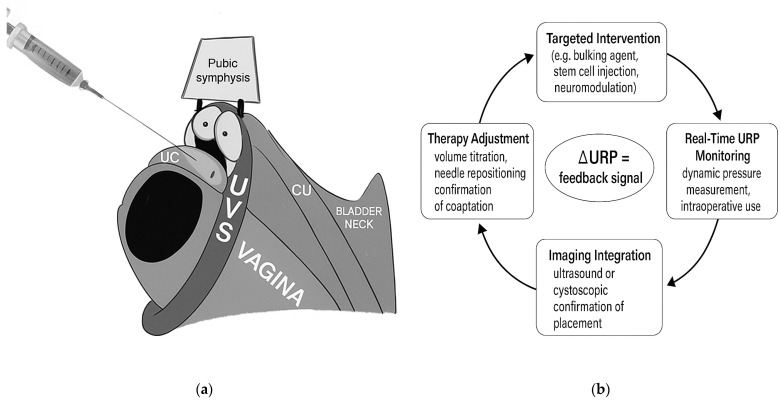
(**a**) Infographic of injection of the components of the distal EUS complex to treat SUI. (**b**) Clinical feedback loop: ΔURP as a procedural feedback signal for targeted therapy, enabled by a machine learning digital platform. The injection is guided by realtime feedback of ΔURP. The resistance change can be correlated with morphological information derived from ultrasound or endoscopy. The platform can direct volume titration and whether the injection is raising the opening pressure.

In this paradigm, ΔURP—the change in resistance from baseline—is the meaningful outcome, not an absolute pressure value. Even modest increases may show successful engagement of the closure mechanism, particularly when paired with anatomic targeting via cystoscopy or ultrasound. This shifts URP from a discarded diagnostic metric to a functional feedback tool, capable of guiding therapy and enabling real-time responsiveness. While there may not be a “normal” URP, changes in its value may correlate well with clinical outcomes. Figure 3 represents how the feedback from ΔURP can direct treatment.

This is particularly relevant in the evolving landscape of stress urinary incontinence (SUI) management. Interest in EUS-targeted therapy is resurgent, as clinicians look for safer, less invasive options with fewer complications than synthetic slings. For instance, bulking agents—traditionally injected at the bladder neck—are now being re-evaluated for distal placement. When injected directly into the EUS under endoscopic guidance, their potential for durable coaptation improves [42]. However, real-time confirmation has been lacking. URP could provide that feedback: a measurable increase in resistance to confirm engagement of the sphincter mechanism [43].

Stem cell–based approaches, such as myoblast or mesenchymal stromal cell injections, are on the horizon to restore EUS integrity. Yet, most trials rely on symptom scores and post hoc urodynamic data, lacking intraoperative endpoints. Incorporating URP as a functional biomarker—measured before and after injection—could offer immediate insight into therapeutic effect. Even before histologic engraftment occurs, functional coaptation may already be taking place. Schmid et al. (2021) [44] note that the absence of real-time intraoperative feedback is a major limitation of current regenerative approaches. The integration of physiologic tools like URP may improve the accuracy and predictability of outcomes [44].

Neuromodulatory interventions, including sacral nerve stimulation or pelvic floor retraining, may also receive help from URP monitoring. Although these therapies act through neurologic pathways rather than localized injection, any downstream improvement in distal urethral resistance can be assessed using dynamic URP. In such cases, URP could serve as a noninvasive tracking tool across treatment sessions, revealing gradual improvements in sphincteric function over time.

## 4. Future Clinical Applications

### 4.1. Intraoperative Biomarker

As therapy for stress urinary incontinence (SUI) increasingly targets the external urethral sphincter (EUS), updated diagnostic and procedural tools are urgently needed. Reimagined as a procedural biomarker, urethral retro-resistance pressure (URP) shifts from a static measurement to a real-time physiologic readout—allowing clinicians to assess treatment efficacy during bulking injections, regenerative therapies, or neuromuscular retraining. The concept of ΔURP—the change in resistance from baseline levels—provides an immediate, quantifiable signal of EUS engagement that can be paired with imaging modalities such as cystoscopy or ultrasound. The possibility exists for an integrative real-time model for EUS function.

The logistics of performing URP in a procedural setting will require optimization. Any occlusive interface—such as a soft cone or balloon-tip mechanism—can serve to eliminate retrograde fluid escape, allowing for pressure buildup in the space proximal to the meatus and distal to the sphincter. This zone becomes a dynamic measurement chamber. However, individual variations in urethral anatomy—including patulousity, length, and the spatial relationship between the meatus and the sphincter—will demand device adaptability and procedural innovation to ensure reliable measurements across patients.

### 4.2. Diagnostic Benchmark

Finite element models of the female urethra have highlighted the importance of structure–function relationships in continence. Attari et al. demonstrated how tissue mechanics, collagen distribution, and urethral geometry contribute to pressure transmission and closure forces [45]. These findings suggest that URP may offer a more anatomically and functionally specific measure of distal sphincter resistance than global urodynamic metrics such as leak point pressure (LPP) or maximum urethral closure pressure (MUCP).

A recent ICI-RS consensus report further reinforced these concerns. Chermansky et al. (2024) [46] advocate for a renewed focus on functional female urethral disorders, including high-tone non-relaxing sphincter and urethral instability—conditions often overlooked by standard urodynamic tests. The authors critique the limitations of traditional tools like urethral pressure profilometry (UPP), electromyography (EMG), and LPP, and call for diagnostic methods that better capture dynamic neurophysiologic behavior. Their proposed shift toward real-time, reflexive metrics directly supports the reinterpretation of URP as a feedback tool for assessing distal sphincter coaptation and responsiveness [46].

### 4.3. Artificial Intelligence

The use of AI for waveform analysis is an increasing reality in fields such as cardiac and critical care, where real-time biofeedback is essential information. Measured hundreds of times per second, routine vital sign waveform monitoring provides the immediate ability to detect changes in a patient’s status, such as an alteration in cardiac rhythm, or the development of life-threatening abnormal vital signs requiring immediate intervention [47]. Predictive modeling and tracking responses to therapeutic interventions can be integrated into clinical decision support software (CDSS), which can guide the clinicians actions [48].

The integration of AI-assisted signal interpretation could similarly enhance the precision, consistency, and clinical adoption of URP. As artificial intelligence has been applied to pressure-flow studies and bladder diaries, similar approaches could be used to analyze URP waveforms—automating ΔURP detection, improving inter-operator reliability, and delivering real-time decision support [49]. This transformation could elevate URP from an analog technique to a digitally enabled platform for precision continence care.

### 4.4. Standardizing Continence Outcomes

The adoption of innovation in any clinical field depends on the ability to compare new technologies to established ones using standardized outcomes. In the field of urinary incontinence, however, objective physiologic metrics remain limited. Current assessments rely heavily on subjective measures—such as validated questionnaires—or crude objective tools like pad weight tests and visual cough assessments. These methods, while useful, fail to capture the nuanced physiologic engagement of sphincteric structures during intervention.

Dynamic URP may offer a solution. Although midurethral slings and proximal urethral injections have historically shown little change in URP post-procedure, this likely reflects their distance from the distal sphincteric zone rather than being a limitation of the method itself. In contrast, when URP is applied dynamically—measuring changes in pressure in real time during targeted therapy—it has the potential to directly evaluate EUS engagement.

Clinically, dynamic URP fills a critical diagnostic and procedural gap. It provides targeted, intra-procedural feedback during EUS-directed interventions—something neither multichannel urodynamics nor conventional imaging can currently offer. As therapeutic interest grows in distal coaptation techniques such as stem cell therapy, regenerative bulking, and neuromodulatory approaches, so too will the need for real-time confirmation of functional response. Dynamic URP offers a practical way to close the loop between anatomy, intervention, and physiologic outcome.

## 5. Conclusions

Innovation in urologic therapies requires physiologic tools to confirm real-time engagement of the external urethral sphincter (EUS) during intervention. Urethral retro-resistance pressure (URP), once sidelined for its limitations as a diagnostic test, may deserve renewed attention—not in its original, static form, but reimagined as a real-time, intra-procedural feedback mechanism—bridging the gap between structural targeting and physiologic response.

This paper offers a speculative yet grounded model—a conceptual shift in how we interpret, apply, and develop URP as a procedural adjunct. The logic is straightforward: the EUS is a therapeutic target, and no current tool confirms whether it has been functionally engaged. URP, particularly when reframed as a measure of change (ΔURP), could fill that void. It builds on established anatomy, past pressure-based techniques, and the unmet needs of current procedural workflows.

Future research will need to validate this framework clinically. Direct sphincter injection remains an investigational approach. Yet, without a reliable feedback mechanism, the ultimate value of direct EUS targeting is untestable. By reframing URP as a functional biomarker of EUS integrity, this paper does not present a definitive solution, but proposes a foundation for evidence-based innovation in stress urinary incontinence treatment.

## Data Availability

Not applicable.
